# Supplementary material for: G-CutMix: A CutMix-based graph data augmentation method for bot detection in social networks
Source: PLoS One. 2025 Sep 26;20(9):e0331978. doi: 10.1371/journal.pone.0331978 (PMC12469246; doi:10.1371/journal.pone.0331978)
Supplement: Attachment — Submitted filename: Response to Reviewers.docx [file pone.0331978.s001.docx]

**Manuscript ID:** PONE-D-24-37596

**Article Title: G-CutMix: a CutMix-based Graph Data Augmentation Method for Bot Detection in Social Networks**

**Reviewer#1, Concern#1:** **The reliance on specific hyperparameters, such as the adaptive threshold α, may limit the method's applicability in varying contexts or datasets. Additionally, the paper does not sufficiently address the computational complexity introduced by the G-CutMix method, which could impact its scalability in real-world applications.**

**Author response:** We thank the reviewer for the time and effort of reviewing our manuscript.

**Hyperparameter Sensitivity Analysis:**

In Section 5.2(Parameter Sensitivity Analysis), we expanded our discussion of the hyperparameter α (CutMix ratio). Experiments in Figure 5 demonstrate that α = 0.3 achieves optimal performance across all datasets (MGTAB, Cresci-15, Twibot-20). For instance, on MGTAB, varying α from 0.1 to 0.9 results in accuracy fluctuations within ±1.5%, indicating robustness. We further provide general guidelines for α: For datasets with sparse graphs (e.g., Twibot-20), α ∈ [0.2, 0.4] is recommended. For dense graphs (e.g., MGTAB), α ∈ [0.3, 0.5] yields stable results.

**Computational Complexity Analysis:**

A new subsection, *Computational Complexity*, quantifies the training time of G-CutMix compared to baseline methods. For example: On MGTAB with a GCN backbone, G-CutMix increases training time by 20% compared to vanilla GCN. These results confirm that G-CutMix’s performance gains outweigh its modest computational cost, making it suitable for real-world deployment.

Table 6. Computational complexity comparisons for different methods(s)

| Method | MGTAB | Twibot-20 | Cresci-15 |
| --- | --- | --- | --- |
| GCN | 16.11 | 18.92 | 9.35 |
| SAGE | 18.64 | 21.56 | 10.13 |
| GAT | 20.53 | 24.58 | 12.62 |
| G-CutMix (GCN) | 20.84 | 22.54 | 11.53 |
| G-CutMix (SAGE) | 22.36 | 24.84 | 12.43 |
| G-CutMix (GAT) | 23.62 | 27.15 | 12.98 |

**Reviewer#1, Concern#2:** **Authors could explore other advanced augmentation techniques like GraphSAGE or node feature perturbation. Additionally, techniques such as adversarial training or semi-supervised learning could enhance robustness. The potential of ensemble methods is another avenue not considered.**

**Author response:** We appreciate the reviewer’s suggestions for extending our work. Here are our clarifications:

**GraphSAGE Integration:**

Our framework is backbone-agnostic and explicitly supports GraphSAGE (abbreviated as SAGE in Table 2 and Table 3). For instance, G-CutMix (SAGE) achieves 85.97% accuracy on MGTAB, outperforming vanilla SAGE by 4.87%. This demonstrates that G-CutMix effectively complements existing GNN architectures.

**Adversarial Training and Semi-Supervised Learning:**

While these techniques are promising, they focus on training paradigms (e.g., robustness to perturbations, label efficiency), which are orthogonal to our core contribution: graph-specific data augmentation. We plan to explore this in future work.

**Ensemble Methods:**

The baseline RF-GNN (Table 2) is an ensemble method combining multiple GNNs. G-CutMix(GAT) outperforms RF-GNN by 1.71% F1 on Twibot-20, showing that our augmentation strategy alone achieves competitive results without ensemble overhead.

**Reviewer#1, Concern#3:** **The method's effectiveness is contingent on the quality of the shuffled graph, which may introduce noise. The impact of this should be explored.**

**Author response:** We clarify that the shuffled graph generation process is structure-preserving (Definition 1), ensuring isomorphism with the original graph. To validate this: Ablation Study (Table 5): Removing the shuffle module ("w/o shuffle") degrades performance on MGTAB by -2.02% accuracy (GCN) and -1.65% (GAT), proving its necessity for diversity.

**Reviewer#1, Concern#4:** **G-CutMix primarily focuses on user relationships, potentially overlooking content analysis or user behavior patterns.**

**Author response:** Our framework explicitly incorporates behavioral and content features: For MGTAB, we use 20 user attributes (Section 4.1 Dataset), including: Behavioral: Post frequency, follower/following ratios, account age. Content: BERT embeddings of tweets, description length, verified status. On Twibot-20, we include 17 attributes such as profile metadata and tweet semantics. These features are concatenated with graph embeddings before classification, ensuring a holistic representation Section 3.3 (Node Cutmix Module).

**Reviewer#1, Concern#5:** **Lack of graph structure impact analysis and overfitting concerns.**

**Author response:**

**Graph Structure Analysis:**

Section 5.3 (CutMix for Heterogeneous Graphs) evaluates multi-relation graphs (Table 4). For example, G-CutMix+RGAT achieves 80.69% accuracy on Twibot-20 using both follower and friend relations, proving adaptability to complex structures.

**Overfitting Mitigation:**

Our shallow GNN design (2 layers) and early stopping (200 epochs) prevent overfitting. Validation curves show stable accuracy, with <1% gap between training and test performance. G-CutMix inherently diversifies training data, acting as a regularizer. For instance, on Cresci-15, G-CutMix reduces validation loss by 15% compared to vanilla GCN.

**Reviewer#1, Concern#6:** **Structural issues: misplaced figures, insufficient literature review, hyperparameter clarity.**

**Author response:**

**Expanded Literature Review:**

The Related Work section now cites 12 additional papers, including recent advances in graph augmentation (e.g., GraphMix, G-Mixup) and bot detection (e.g., BotRGCN, SATAR). We also contrast G-CutMix with MixupForGraph, highlighting its advantages in preserving graph locality (Section 2.3 Data Augmentation).

**Supplementary experimental analysis:**

The consistent superiority of G-CutMix-enhanced models, average 1.91% F1 improvements on MGTAB, 1.95% on Cresci-15, 3.82% on Twibot-20 stems from its ability to synergize follower and friend relations. Unlike baseline RGCN/RGAT that process relations sequentially, our method’s isomorphic shuffling and feature fusion create implicit cross-relational attention—for instance, amplifying signals where follower-friend reciprocity indicates coordinated bot behavior. While both RGCN and RGAT benefit from G-CutMix, the greater improvements with RGAT highlight our method’s compatibility with attention mechanisms. The learnable merging weights in G-CutMix likely synergize with RGAT’s edge-specific attention, enabling adaptive reweighting of mixed features.

The ablation study results in Table 5 reveal critical interdependencies between G-CutMix's components and dataset characteristics. The most pronounced performance degradation occurs when removing the Attribute Connection Module (average 3.8% F1 drop across datasets), particularly severe in Twibot-20 (6.2% accuracy decline for GCN), suggesting that social bots' attribute camouflage strategies - such as profile metadata manipulation - require explicit attribute correlation modeling to detect. Interestingly, while Node Shuffle removal impacts MGTAB most significantly (1.5-2.5% accuracy reduction), its effect diminishes in Twibot-20 where temporal behavioral patterns dominate, implying that structural isomorphism preservation becomes less critical when bots exhibit strong activity sequence signatures. These findings collectively demonstrate that G-CutMix's power emerges from the synergistic combination of its components rather than any single module.

**Misplaced figures:**

We have systematically readjusted the placement of figures and tables throughout the article to enhance the overall logical flow and visual consistency.

**Reviewer#2, Concern#1:** **This manuscript is lack of core novelty and overall poorly presented.**

**Author response:** We respectfully disagree but acknowledge the need to clarify our contributions:

**Novelty:**

G-CutMix is the first method to adapt CutMix to graph-structured data for bot detection. Unlike image-based CutMix, we propose: Isomorphic Graph Shuffling to preserve structural integrity (Section 3.2). Attribute Connection Module to retain original node features post-mixing (Section 3.3). These innovations are validated by outperforming MixupForGraph by 3.2% F1 on Twibot-20 (Table 2).

**Reviewer#2, Concern#2:** **Proposed section is not enough as per the standard of this journal. In addition, the results are not presented well and lack of validations.**

**Author response:** Your comment is very important to us.

To our knowledge, G-CutMix is the first to adapt CutMix for graphs by combining feature mixing with isomorphic shuffling, addressing irregular graph structures.

**Added Recent Works:**

We have added seven state-of-the-art references (e.g., BotRGCN, SATAR, and BotCL) to systematically integrate recent breakthroughs in graph neural network-based detection methodologies. The restructured introduction now establishes a coherent narrative flow, beginning with fundamental challenges in bot detection, progressing through critical limitations in existing approaches, and concluding with our novel technical framework.

**Supplementary discussion：**

We have supplemented background and motivation and the discussion of the t-SNE plot results, improved the discussion of the results in Tables 4 and 5, and added and explained the experiments on computational complexity.

**Reviewer#3, Concern#1:** **The proposed G-CutMix method effectively enhances GNN training by introducing a node mixing technique, particularly excelling in scenarios with small datasets, which is a commendable technical innovation. However, when compared with existing augmentation methods (e.g., MixupForGraph), the authors are encouraged to further analyze the theoretical advantages of G-CutMix beyond the experimental performance improvements.**

**Author response:** Thank you for highlighting this crucial aspect. We have expanded the theoretical analysis in Section 3.1 (Background and Motivation) to clarify the unique advantages of G-CutMix:

**Locality Preservation via Binary Masking:**

Unlike MixupForGraph, which linearly interpolates node features, G-CutMix employs region-level feature swapping using a binary mask M (Equation 1). This ensures that local structural patterns (e.g., community-specific interactions) are preserved during augmentation, whereas linear interpolation may blur such patterns. For example, in social graphs, bots often exhibit localized behavioral anomalies (e.g., sudden spikes in follower requests). By retaining intact feature regions, G-CutMix helps GNNs capture these subtle signals more effectively.

**Reviewer#3, Concern#2:** **The tabular results are clear, but the visualizations (e.g., t-SNE plots) would benefit from detailed explanations of the differences in distributions across methods to enhance their persuasive power.**

**Author response:** Your comment is very important to us.

**Supplementary discussion：**

According to the reviewer's suggestion, the following supplements are discussed about the results of t-SNE plots: “The embeddings obtained by the GCN model exhibit the highest degree of overlap in the feature space, as evidenced by the fact that most green points are occluded by orange points. DropEdge and MixupGCN demonstrate better distinguishability in the feature space compared to GCN. The embeddings generated by CutMix show the lowest overlap in the feature space and achieve the highest distinguishability.”

**Reviewer#3, Concern#3:** **The description of the methodology is detailed, but certain formulas (e.g., the choice of α in CutMix) and parameter settings (e.g., the edge dropout rate in DropEdge) lack a thorough explanation of their specific impact on performance. It is recommended to supplement experiments or analyses to increase the rigor of the descriptions.**

**Author response:** Your comment is very important to us.

In Section 5.2 (Parameter Sensitivity Analysis), we expanded our discussion of the hyperparameter α (CutMix ratio). Experiments in Figure 5 demonstrate that α = 0.3 achieves optimal performance across all datasets (MGTAB, Cresci-15, Twibot-20). For instance, on MGTAB, varying α from 0.1 to 0.9 results in accuracy fluctuations within 1.5%, indicating robustness. We further provide general guidelines for α: For datasets with sparse graphs (e.g., Twibot-20), α ∈ [0.2, 0.4] is recommended. For dense graphs (e.g., MGTAB), α ∈ [0.3, 0.5] yields stable results.

Based on the reviewers' suggestions, we have supplemented the description of DropEdge parameters in this manuscript.

**Reviewer#3, Concern#4:** **The structure of the paper is clear, and the language is precise, but some sections (e.g., the literature review in the introduction) could be further streamlined to improve the overall flow of the paper. Overall, this paper proposes a novel and effective graph data augmentation method with a certain degree of technical innovation, and its effectiveness is verified through comprehensive experiments. If the theoretical analysis and experimental discussions are supplemented and improved, the contribution of the paper will be more significant.**

**Author response:** Your comment is very important to us. We restructured the Introduction to enhance clarity and conciseness:

**Added Recent Works:**

Incorporated 7 new references (e.g., BotRGCN, SATAR , BotCL) to reflect the latest advances in GNN-based bot detection. The revised introduction now progresses logically from problem statement to technical gaps and our solution.
